# Cobb Syndrome Manifesting as Repetitive Seizures in a 10-Year-Old Girl: A Case Report and Literature Review

**DOI:** 10.3389/fneur.2019.01302

**Published:** 2019-12-06

**Authors:** Lin Wan, Wen-Rong Ge, Xiu-Yu Shi, Jing Wang, Lin-Yan Hu, Li-Ping Zou, Guang Yang

**Affiliations:** ^1^The First Medical Center of Chinese PLA General Hospital, Beijing, China; ^2^Beijing Friendship Hospital, Capital Medical University, Beijing, China

**Keywords:** Cobb syndrome, seizure, subarachnoid hemorrhage, diagnosis, headache

## Abstract

Cutaneous vertebral medullary angiomatosis, also known as Cobb syndrome, is a rare segmental neurocutaneous syndrome. This syndrome is considered to be a non-hereditary congenital disease that is usually associated with arteriovenous malformations in the skin and spine. The clinical manifestations are complex because the lesions can involve the spine, spinal cord, skin, and even the viscera. Here, we present the case of a 10-year-old girl who was admitted to hospital due to headache with two episodes of convulsions. Previous examination at another hospital found no evidence of any abnormalities on either cranial or intracranial vascular magnetic resonance imaging (MRI). However, the patient had a history of subcutaneous hemangioma. Following exhaustive tests at our hospital, she was diagnosed with Cobb syndrome. She received surgery, treatment for decreasing intracranial pressure, and hormonal and nutritional support. She subsequently remained stable, with no recurrence of convulsions over a 9-year follow-up period. Here, we expand upon the clinical manifestations of Cobb syndrome and propose mechanisms for the underlying pathogenesis. We hope that our experience can help avoid missed diagnoses and misdiagnosis and provide more clinical evidence for early diagnosis.

## Introduction

**Figure 1:**
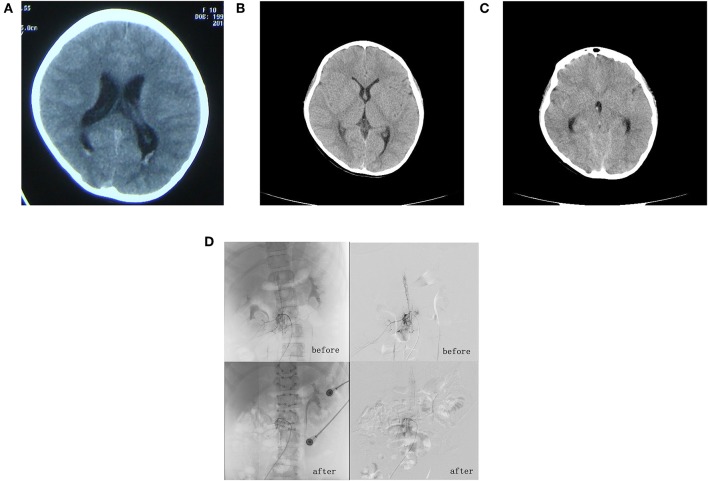
**(A)** First craniocerebral computed tomography (CT) scan, taken 3 months prior to admission. This revealed a full ventricular system with a significant bilateral subependymal vascular shadow in the posterior horn of the ventricles. **(B)** Second craniocerebral CT scan, taken 2 days after admission, which failed to identify any sign of significant ventricular and subarachnoid hemorrhage. **(C)** Third craniocerebral CT scan, taken 5 days after admission. This revealed mild ventricular and subarachnoid hemorrhage. **(D)** Angiography of the entire spine revealed a spinal dural arteriovenous fistula involving segments T11–L2.

A 10-year-old girl was admitted to our hospital due to two episodes of seizures within the previous 3 months. Prior to admission, the patient presented with a generalized tonic-clonic seizure that was followed by a headache of unknown cause. One day later, the patient experienced lumbodorsal pain without fever or diarrhea. Cranial magnetic resonance imaging (MRI) and intracranial arterial nuclear magnetic angiography (MRA), carried out previously in another hospital, failed to identify any abnormalities. Symptoms were alleviated following the administration of dexamethasone and the reduction of intracranial pressure. Three days prior to admission, the patient had experienced pharyngalgia, without fever, vomiting or diarrhea. Later that day, she had experienced a second seizure which was similar to the first episode; this occurred during the night and was followed by headache, vomiting and rigidity in the neck. Her head was subsequently scanned by computed tomography (CT) in a local hospital, which revealed ventriculomegaly and showed that prominent subependymal vessels in the posterior horn of the bilateral ventricles (Figure 1A). No obvious abnormalities were detected by way of hemogram, blood biochemistry or blood gas analysis. However, the patient still presented with headache after the administration of cefmenoxime, mannitol, and ibuprofen. Therefore, the patient was transferred to our hospital for further evaluation. The patient was born at term with a spontaneous delivery and normal APGAR score. No abnormalities had occurred during the perinatal period and there was no family history.

## Case Presentation

Upon admission at our hospital, there was no clinical history to note except that she had undergone ultrasound examination when she was 6-years-old due to a right inguinal mass; this was later diagnosed as a hemangioma but no special treatment was provided.

A physical examination showed a palpable 3 × 3 cm right inguinal mass without tenderness or movement and neck rigidity; muscle strength and tension were normal. The patient was positive for the Kernig reflex and Brudzinski reflex but was bilaterally negative for the Babinski reflex. We performed a CT scan of the skull but there was no evidence of significant intraventricular and subarachnoid hemorrhage (Figure 1B). We also carried out an ultrasound examination, which showed a large honeycomb-shaped anechoic area in the right iliopsoas adjacent to the external iliac vein and inguinal area; this was considered to be the hemangioma that was identified previously. Upon admission, we also performed lumbar puncture. The pressure of the cerebrospinal fluid was 1.5 kPa (150 mm H_2_O). Three tubes of cerebrospinal fluid were collected; all showed light hemic cerebrospinal fluid. A electroencephalogram examination was carried out; this showed a 130–280 μV/3–4 Hz high amplitude theta rhythm in the right occipital region and right posterior temporal areas. By referring to the results of the cerebrospinal fluid examination, we were able to exclude infection of the central nervous system. Five days after admission, the patient felt pain in her legs when standing and experienced a generalized tonic-clonic seizure after lying down. Urgent head CT scans revealed a minor hemorrhage in the ventricle and subarachnoid space (Figure 1C). She was immediately transferred to the Department of Neurosurgery at our hospital. Subsequent whole cranial and spinal cord angiography revealed spinal vascular malformations and a malformation vascular mass in the T11–L2 segments, which was considered to be a spinal cord arteriovenous malformation (SCAVM-intramedullary) (Figure 1D). The patient subsequently underwent surgery under general anesthesia. We excised the vascular mass/malformation in segments T11–L2 of the spinal cord malformation; this was followed by laminoplasty and dural repair. The patient regained conscious following the postoperative administration of hemostatic drugs, intravenous fluid therapy and 100 mg of phenobarbital (intramuscular, once daily) for 8 days. Eventually, she was seizure free, physically stable, and was subsequently discharged on day 35. At the time of discharge, she was diagnosed with Cobb syndrome related to vascular arteriovenous malformation of the spinal cord, complicated by a subcutaneous soft-tissue hemangioma. Over a 9-year follow-up period, the patient was regularly assessed via telephone interview; there were no reports of any more seizures. A review of the whole cranial and spinal cord angiography revealed that the fistulas had clearly shrunk and that there was no nidus (Figure 1D). The patient and her parents were informed about the intentions of this study and signed an informed consent form; they also agreed for us to publish this manuscript. This study was approved by the Ethics Committee of First Medical Center of PLA General Hospital, China (No. 2018267).

## Discussion

Cobb syndrome, also known as cutaneous vertebral medullary angiomatosis, is generally considered to be a non-hereditary congenital disease. This syndrome is characterized by segmental vascular malformation involving the skin, soft tissue, spine, spinal cord, and even the viscera of the same body segment. The first description of this syndrome was reported by Berenbruch in 1890 but it was not until 1915, when Cobb syndrome was first reported in an 8-year-old boy with flaccid paralysis (1), that there started to be noticeable recognition of Cobb syndrome. This disease predominantly occurs in children and adolescents; there are no obvious differences in relation to gender or race. Very few case reports have been published featuring patients with Cobb syndrome (1–4). This is due to our limited understanding of this syndrome and its clinical manifestations. Segments T3–T9 are usually the most susceptible segments (5). Cobb syndrome is now diagnosed when patients experience three or more of the following five factors (6): (1) intramedullary vascular malformation; (2) intraspinal epidural hemangioma; (3) vertebral hemangioma; (4) paravertebral hemangioma; or (5) cutaneous or subcutaneous hemangioma, brown nevi or patchy coffee spots (7). Interventional embolization and surgical resection are the main forms of therapy for Cobb syndrome. Our patient was confirmed to have Cobb syndrome because she satisfied three items of the diagnostic criteria listed above. Our patient was subsequently treated surgically and is now stable.

Existing literature suggests that this disease is associated with a subacute or chronic onset (3), typically manifested by sudden root pain in the back and lower extremities below a certain spinal cord level, accompanied by numbness of the skin. Secondary symptoms include weakness in the lower extremities and urinary dysfunction (3, 4, 8). Paraplegia and sensory disturbance are common in patients with acute onset and permanent sequelae can develop if the syndrome is missed or misdiagnosed (3). These sequelae can be avoided by early diagnosis and treatment based on clinical manifestations. Our patient was different than those reported in the existing literature because she presented with headache and convulsions as the first manifestations. She also had a history of hemangioma. She showed no increase in intracranial pressure at first onset. Furthermore, MRI of the brain and intracranial vessels during outpatient visits failed to identify any abnormalities, although spinal angiography was not carried out. An increase in intracranial pressure was observed during the first episode of seizure prior to admission; CT examination also showed that the cranial ventricles had filled simultaneously. Following hospital admission, there was no evidence of hemangioma on the surface of the skin. With regards to the nervous system, Kernig's sign and Brudzinski's sign were both positive, but no typical signs of sensory disorder of the spinal cord were evident. After admission, the convulsive seizures were accompanied by signs of craniocerebral stimulation, but there was no obvious sensory impairment or symptoms of paralysis.

The etiology of convulsions is highly complex. Previous studies have reported that subarachnoid hemorrhage can cause seizures, and that the pathogenesis of this condition may be related to direct or indirect chemical effects, all of which may reduce the onset threshold of seizures at different time points (9). In our present case, CT scans performed in the outpatient department were indicative of ventricular expansion. However, these findings were not apparent when the patient was scanned again following hospital admission. However, we did successfully identify intraventricular and subarachnoid hemorrhage. All three tubes of cerebrospinal fluid taken by lumber puncture were hemorrhagic, thus excluding puncture injury (suggestive of subarachnoid hemorrhage). Angiography of the entire spinal cord suggested that blood had leaked into the cerebrospinal fluid circulation in the subarachnoid space following the rupture and hemorrhage of an aneurysm, thus leading to an increase in cranial pressure. We suspect that the seizure was caused by the joint action of high intracranial pressure and stimulation by blood. Comparisons of multiple CT scans before and after clinical events were highly useful in supporting our clinical decisions. Following surgery, our patient experienced no further convulsions for the next 9 years of follow-up.

Herein, we reported a case of Cobb syndrome that was associated with clinical symptoms that have not been previously described. The main symptoms experienced by our patient were subcutaneous hemangioma and headache accompanied by epileptic seizures. Our findings therefore provide further enrichment regarding the clinical phenotype of Cobb syndrome. We recommend that clinicians should consider the possibility of Cobb syndrome in patients who experience subcutaneous or cutaneous hemangioma and headache accompanied by epilepsy, even in the absence of sensory disturbance or paralysis. In such cases, angiography of the entire spine is particularly important.

## Data Availability Statement

All datasets generated for this study are included in the article/supplementary material.

## Ethics Statement

The studies involving human participants were reviewed and approved by Ethics Committee of First Medical Center of PLA General Hospital, China. Written informed consent to participate in this study was provided by the participants' legal guardian/next of kin. Written informed consent was obtained from the individual(s), and minor(s)' legal guardian/next of kin, for the publication of any potentially identifiable images or data included in this article.

## Author Contributions

W-RG and LW wrote the first draft of the manuscript. W-RG, LW, X-YS, JW, L-YH, L-PZ, and GY wrote sections of the manuscript. All authors made a substantial, direct and intellectual contribution to the work, and approved the final version for publication.

### Conflict of Interest

The authors declare that the research was conducted in the absence of any commercial or financial relationships that could be construed as a potential conflict of interest.
